# Antineutrophil Cytoplasmic Antibody Frequency in Chronic Hepatitis B Patients

**DOI:** 10.1155/2014/982150

**Published:** 2014-08-05

**Authors:** Turan Calhan, Abdurrahman Sahin, Resul Kahraman, Mustafa Erhan Altunoz, Fatma Ozbakır, Kamil Ozdil, Hacı Mehmet Sokmen

**Affiliations:** ^1^Department of Gastroenterology, Umraniye Education and Research Hospital, 34764 Istanbul, Turkey; ^2^Department of Gastroenterology, Haydarpasa Numune Education and Research Hospital, Istanbul, Turkey; ^3^Central Research Laboratory, Cerrahpasa Medical Faculty, University of Istanbul, Istanbul, Turkey

## Abstract

*Background*. Chronic hepatitis B (CHB) is a viral disease, common across the world, and associated with several extraintestinal manifestations including vasculitis. Antineutrophil cytoplasmic antibodies (ANCAs) are sensitive and specific markers for vasculitides. There is limited data available in the literature on whether ANCA formation is stimulated by CHB infection. In the present study we aimed to identify the frequency of ANCA in CHB patients. *Methods*. A total of 174 subjects were included in the study (87 CHB patients, 87 control subjects). Perinuclear-ANCA (P-ANCA), cytoplasmic-ANCA (C-ANCA), myeloperoxidase ANCA (MPO-ANCA), and proteinase 3-ANCA (PR3-ANCA) were studied. IFA was used for P-ANCA and C-ANCA assays, and ELISA was used for MPO-ANCA and PR3-ANCA assays. *Results*. ANCA positivity was high in both groups (31% in the CHB group and 26% among controls). There were no significant differences between the groups for P-ANCA and MPO-ANCA (*P* = 0.6 and *P* = 0.6, resp.). Frequency of borderline positive C-ANCA and all positive PR3-ANCA (positive + borderline positive) was significantly higher in the CHB group (*P* = 0.009 and *P* = 0.005, resp.). *Conclusions*. In the present study, the frequency of ANCA was high in both groups. The CHB group had a relatively higher frequency of ANCA positivity compared to controls. Borderline positive C-ANCA and positive PR3-ANCA were significantly higher in the CHB group. These results suggest that ANCA may have a high prevalence in Turkey. Patients with CHB should be evaluated particularly for C-ANCA and PR3-ANCA in the presence of vasculitic complaints and lesions.

## 1. Introduction

Chronic hepatitis B (CHB) is a major cause of morbidity and mortality worldwide. Approximately 400 million individuals are infected with hepatitis B virus (HBV) globally [1, 2] and 1 million patients die annually due to HBV-associated complications, such as liver cirrhosis and hepatocellular carcinoma [3, 4]. CHB infection is associated with several extrahepatic manifestations, some of which are systemic (e.g., serum sickness and polyarteritis nodosa) and some are localized, such as dermatologic manifestations (e.g., urticaria, leukocytoclastic vasculitis, and essential mixed cryoglobulinemia) [5, 6]. It has been demonstrated that antineutrophil cytoplasmic antibody (ANCA) positivity could be detected in many cases of vasculitis associated with viruses. These viruses are involved in the etiopathogenesis of ANCA positive vasculitis [7–15].

Antineutrophil cytoplasmic antibodies (ANCAs) are autoantibodies, usually of IgG structure, formed against azurophilic granules and lysosomal components in the cytoplasm of neutrophils and monocytes [16]. ANCAs are sensitive and specific markers of ANCA-related vasculitis and their frequency in the normal population is estimated to be low (<5%) [17, 18].

It is controversial whether vasculitis or other immune related presentations are related to ANCA in CHB patients. Moreover, it is important to determine whether HBV stimulates ANCA formation without triggering vasculitis or other immune related manifestations. There is insufficient data about this issue in CHB patients. We aimed in the present study to identify the frequency of perinuclear-ANCA (P-ANCA), cytoplasmic-ANCA (C-ANCA), myeloperoxidase-ANCA (MPO-ANCA), and proteinase 3-ANCA (PR3-ANCA) subgroups of CHB patients in comparison with healthy controls.

## 2. Materials and Methods 

This was a cross-sectional observational cohort study. From September 2011 to February 2012, 87 consecutive chronic hepatitis B patients (male/female: 54/33; mean age: 36 ± 11 years) who attended the hepatology outpatient clinic for routine followup were enrolled as the study population. 87 gender and age matched healthy individuals (male/female: 44/43; mean age: 37 ± 11 years) were enrolled as the control group. The inclusion criteria for the CHB group were patients with HBsAg positivity persisting for more than 6 months, patients above 16 years of age, patients without complications such as cirrhosis or hepatocellular carcinoma, patients without CHB-related extrahepatic manifestations, patients with negative HCV and HIV tests, and patients without any malignancy and/or systemic disease (e.g., diabetes mellitus, hypertension, cerebrovascular disease, cancer, autoimmune disease, and rheumatic disease). Inclusion criteria for healthy controls were individuals above 16 years of age with no systemic disease who tested negative for complete viral hepatitis serology including anti-HBc IgG and hospitalized for other reasons, such as dyspeptic symptoms or volunteering hospital staff satisfying the same criteria. Individuals with high erythrocyte sedimentation rate and C-reactive protein measurement were excluded from either of the groups in the study. Demographic data, duration of disease, serum liver transaminase levels, HBV-DNA levels, HBeAg status, and antiviral treatment status for the CHB group were noted. Serum samples were obtained from all participants and kept frozen at −80°C until the study date. Written informed consent was obtained from all patients. The study protocol was approved by the local ethics committee of our hospital (no. 14066, 23.09.2011).

P-ANCA and C-ANCA were analyzed with an indirect Immunofluorescence Assay (IFA) (ImmuGlo—IMMCO Diagnostic, Buffalo, NY, USA). MPO-ANCA and PR3-ANCA were analyzed with micro-ELISA (ImmuLisa—IMMCO Diagnostic, Buffalo, NY, USA). Results for IFA were categorized as negative, borderline positive, and positive. For ELISA, results less than 10 EU/mL were considered negative, results between 10 and 12.5 EU/mL were considered borderline positive, and results above 12.5 EU/mL were considered positive.

In this study, assuming that there would be a difference of 19% between the two groups for ANCA frequency, the sample size required to detect 80% power at *α* = 0.05 level required at least 80 subjects for each group. For statistical analyses, NCSS (Number Cruncher Statistical System) 2007 and PASS (Power Analysis and Sample Size) 2008 Statistical Software (Utah, USA) were used. Quantitative data with normal distribution was analyzed with Student's *t*-test and data without normal distribution was analyzed with Mann-Whitney *U* test. Chi-square and Fisher's exact tests were used to compare qualitative data. Statistical significance was set at *P* < 0.05 with a 95% confidence interval.

## 3. Results

A total of 174 subjects were enrolled in the study. The study group and the control group did not differ significantly by age or gender. Comparison of the two groups for ANCA positivity (total ANCA positivity) did not yield any significant differences. Age, gender, and ANCA positivity information of the groups are provided in Table 1.

Comparisons of the two groups for positive, borderline positive, and any (all) positive (positive + borderline positive cases) results for P-ANCA and MPO-ANCA did not reveal significant differences. Frequency of borderline positive C-ANCA and of any (all) positive PR3-ANCA (positive + borderline positive cases) was significantly higher in the CHB group (*P* = 0.009 and *P* = 0.005, resp.). Detailed comparative ANCA data of the two groups are given in Table 2.

In the CHB group, the mean disease duration was 7 ± 4 years, mean aspartate transaminase (AST) level was 30 ± 20 I/U, mean alanine transaminase (ALT) level was 45 ± 47 I/U, 84% (73/87) of the patients in this group were HBeAg negative, and 56% (49/87) had HBV-DNA levels of ≤2000 IU/mL. Among these, 39% (34/87) were receiving antiviral therapy (12 with entecavir, 19 with tenofovir, and 3 with lamivudine), while 61% (53/87) were not receiving antiviral treatment. ANCA positive cases in the CHB group were compared for disease duration, HBeAg positivity, antiviral therapy, and HBV-DNA levels, yielding no significant relationship.

## 4. Discussion

The role of hepatotropic viruses such as HBV and hepatitis C virus (HCV) with similar immunopathogenesis in ANCA development and ANCA-related vasculitis etiology is a current topic of investigation. Available data on this topic is insufficient and further research is necessary. In a study of chronic viral hepatitis patients, the frequency of ANCA was higher in an HCV group compared to controls although the same was not observed for the HBV group [19]. A study conducted on HCV patients suggests that PR3-ANCA positivity was more common and that HCV could be a cause for ANCA-related vasculitis [20]. In two case reports of Wegener granulomatosis patients with hepatitis C, one was positive for MPO-ANCA [21], while another patient had a high titer of PR3-ANCA positivity [22]. In both reports the role of HCV in systemic vasculitis was underlined. Another case presentation discussed concurrent Churg-Strauss syndrome and HBV in an ANCA positive patient [11].

In this study, 31% of the patients (27/87) in the CHB group had ANCA positivity (any ANCA positivity). These results demonstrate a high prevalence of ANCA positivity in the CHB group. The most significant difference between CHB and control groups arises from borderline positivity for C-ANCA and PR3-ANCA (for C-ANCA: 11 with borderline positive versus 2 in controls; for PR3-ANCA: 12 with borderline positive versus 5 in controls). The borderline positivity in the CHB group might be the result of the patient selection criteria. The patients with rheumatic manifestations and elevated inflammatory markers were excluded. Therefore the remaining patients were the ones with borderline positivity with subclinical manifestations. In other words, the higher number of borderline positive subjects in our study could suggest that CHB stimulates ANCA development without exhibiting clinical manifestations.

ANCA is divided into three patterns by indirect immunofluorescence: C-ANCA, P-ANCA, and atypical ANCA. Classical P-ANCA occurs with antibodies directed to MPO. P-ANCA without nuclear extension occurs with antibodies to bacterial permeability increasing factor, cathepsin G, elastase, lactoferrin, and lysozyme. The most common antigens used on an ELISA are MPO and PR3, so we investigated MPO and PR3 subgroups in the present study [23]. We found a higher positivity frequency only for both C-ANCA and PR3-ANCA patterns in CHB patients. Taking into account our results, it is suggested that CHB may cause development of antigens that influence cytoplasmic ANCA patterns.

The pathogenesis of virus-related vasculitides is thought to be heterogeneous and at least two mechanisms have been suggested to be involved; in the first mechanism, viral replication induces direct damage to the blood vessel wall leading to viral arteritis, and the second is a theory that suggests that vascular damage could be associated with the immune mechanism [24]. Immunologic mechanisms that are involved in the pathogenesis may be related to cellular and/or humoral immunity and may be in the form of immunocomplex deposition [25, 26]. Extrahepatic manifestations seen in HBV are believed to result mostly from immunological pathways [6, 27]. It has been suggested that persistent antigen load secondary to chronic infection and high viral replication rate lead to excessive formation of immunocomplexes, and these circulating immunocomplexes deposited in intermediate and small-sized vessels initiate vasculitic processes [28, 29]. Vasculitic lesion development in HBV is believed to be associated rather with the HBeAg antigen or viral replication [30]. In our study, 84% of the patients were HBeAg negative and 56% had HBV-DNA levels below 2000 IU/mL. These findings may account for the lack of significance for high total ANCA, despite the significant rates of positivity identified for C-ANCA and PR3-ANCA in the CHB group. For a better understanding of the ANCA and HBV relationship, studies involving patient groups with high viral load and who are HBeAg (+) could provide valuable information.

Adequate data on CHB and the incidence of ANCA in healthy individuals in Turkey is not available. The high ANCA prevalence in the healthy control group (26%) was a remarkable finding of our study. In both groups, positive samples for ANCA were studied at least two times. Samples were then studied for antinuclear antibody (ANA). After the proven negative results for ANA, the sample was considered ANCA positive. This ANCA prevalence of 26% in our controls versus the <5% value reported in the literature makes it difficult to determine whether ANCA positivity in CHB patients is due to CHB itself or a higher prevalence of ANCA in the Turkish population. This complicates the assessment of the effect of HBV on ANCA despite the significantly higher rates of PR3-ANCA and C-ANCA.

There are some limitations of this study. Firstly, we did not evaluate atypical ANCA patterns because these patterns constitute approximately 5% of all ANCA, and the most common antigens are PR3 and MPO. Another limitation is that this study was conducted on CHB patients for which more than half were HBe antigen negative. Further studies on HBe antigen positive patient groups are needed. This study is a single center study that was conducted with a small number of patients. Thus there is also a need for a larger series.

## 5. Conclusions

ANCA frequency was high in both groups in our study. The CHB group had a relatively higher number of ANCA positive individuals compared to the control group. Borderline positive C-ANCA and positive PR3-ANCA (positive + borderline positive cases) were significantly higher in the CHB group. These results indicate that patients with CHB infection should be evaluated particularly for C-ANCA and PR3-ANCA in the presence of vasculitic complaints and lesions. However, larger studies with a larger number of subjects are needed to verify these data.

## Figures and Tables

**Table 1:** Comparison of the two groups by age, gender, and ANCA positivity (any ANCA positivity).

	Control (*n* = 87)	CHB (*n* = 87)	*P*
Age, years (mean ± SD)	37.33 ± 11.48	36.99 ± 11.27	0.8

	*n* (%)	*n* (%)	*P*

Gender			
Female	43 (49.4%)	34 (39.1%)	0.1
Male	44 (50.6%)	53 (60.9%)	
ANCA (any ANCA positivity)			
Positive	23 (26.4%)	27 (31%)	0.5
Negative	64 (73.6%)	60 (69%)	

CHB: chronic hepatitis B; ANCA: antineutrophil cytoplasmic antibody; and Mean ± SD: mean ± standard deviation.

**Table 2:** Comparison of the two groups by P-ANCA, C-ANCA, MPO-ANCA, and PR3-ANCA.

	Control (*n*, %)	CHB (*n*, %)	*P*
P-ANCA			
Negative	80 (92%)	78 (89.7%)	—
Positive	6 (6.9%)	3 (3.4%)	0.4
Borderline positive	1 (1.1%)	6 (6.9%)	0.1
All positive (any positivity)	7 (8%)	9 (10.3%)	0.6
C-ANCA			
Negative	78 (89.7%)	71 (81.6%)	—
Positive	7 (8%)	5 (5.7%)	0.5
Borderline positive	2 (2.3%)	11 (12.6%)	**0.009**
All positive (any positivity)	9 (10.3%)	16 (18.4%)	0.1
MPO-ANCA			
Negative	78 (89.7%)	80 (92%)	—
Positive	5 (5.7%)	2 (2.3%)	0.4
Borderline positive	4 (4.6%)	5 (5.7%)	1
All positive (any positivity)	9 (10.3%)	7 (8%)	0.6
PR3-ANCA			
Negative	81 (93.1%)	68 (78.2%)	—
Positive	1 (1.1%)	7 (8%)	0.06
Borderline positive	5 (5.7%)	12 (13.8%)	0.07
All positive (any positivity)	6 (6.9%)	19 (21.8%)	**0.005**

CHB: chronic hepatitis B; ANCA: antineutrophil cytoplasmic antibody; P: perinuclear; C: cytoplasmic; MPO: myeloperoxidase; and PR3: proteinase 3.
